# Intestinal mucus acts as a nutrient source and signal for* Klebsiella pneumoniae*

**DOI:** 10.20517/mrr.2025.112

**Published:** 2026-04-29

**Authors:** Taylor D. Ticer, Pramita Suresh, Subhomitra Ghoshal, Anna M. Tingler, Rachel Stubler, Adelaide E. Horvath, Janiece S. Glover, Terri N. Ellis, Melinda A. Engevik

**Affiliations:** ^1^Department of Microbiology & Immunology, Medical University of South Carolina, Charleston, SC 29425, USA.; ^2^Department of Regenerative Medicine & Cell Biology, Medical University of South Carolina, Charleston, SC 29425, USA.; ^3^Department of Biology & Biochemistry, University of Houston, Houston, TX 77004, USA.; ^4^Department of Mathematics, University of Houston, Houston, TX 77004, USA.; ^5^Department of Biology, University of North Florida, Jacksonville, FL 32224, USA.

**Keywords:** *Klebsiella pneumoniae*, mucus, glycosyl hydrolase, glycans, oligosaccharides, antibiotics

## Abstract

**Background:** *Klebsiella pneumoniae* can colonize the gastrointestinal tract, yet its interactions with intestinal mucus remain poorly defined. In this study, we examined the capacity of *Klebsiella pneumoniae* (*K. pneumoniae*) to adhere and use intestinal mucus and its associated glycans.

**Methods:** Multiple commercial and clinical *K. pneumoniae* isolates were tested for adhesion to porcine and human Mucin 2 (MUC2) using fluorescence-based assays and microscopy. *In vivo* mucus localization was examined in colonized mice by fluorescent *in situ* hybridization (FISH). Genomic analyses of *K. pneumoniae* genomes were performed to identify glycosyl hydrolases and sugar utilization pathways. Growth on mucin-derived monosaccharides or intact mucus was assessed in minimal media. Biofilm formation and aminoglycoside susceptibility were measured in the presence or absence of mucus.

**Results:** All *K. pneumoniae* strains adhered robustly to porcine and human MUC2 *in vitro* and we found *K. pneumoniae* localized to the murine mucus layer *in vivo*. Genomic analysis of over 1,000 *K. pneumoniae* isolates revealed that most strains possess glycosyl hydrolases targeting internal galactose, N-acetyl-glucosamine (GlcNAc), and N-acetyl-galactosamine (GalNAc) glycan sugars, though they lack enzymes to cleave terminal fucose or N-acetyl-neuraminic acid. Consistent with this finding, we found that *K. pneumoniae* alone could not grow in minimal media with intact mucus as a sole carbon source. However, we found that *K. pneumoniae* could grow with free mucus glycan-derived sugars galactose, GlcNAc, GalNAc fucose and N-acetyl-neuraminic acid. Mucus did not alter biofilm formation, but it significantly increased sensitivity to gentamicin, kanamycin and streptomycin.

**Conclusion:** These findings identify mucus as an important modulator of *K. pneumoniae* colonization and antibiotic responsiveness.

## INTRODUCTION

*Klebsiella pneumoniae* (*K. pneumoniae*) is a Gram-negative opportunistic pathobiont that has garnered attention in recent years due to its antibiotic resistance and capacity to cause severe infections^[1]^. It has been implicated in a wide range of diseases, including liver abscesses, pneumonia, urinary tract infections, and bloodstream infection^[2-5]^. The risk of *K. pneumoniae* infection is highest among immunocompromised individuals, neonates, and the elderly^[6-8]^. Although *K. pneumoniae* is not a dominant member of the healthy gut microbiota, it is found at low levels in the gastrointestinal tract and its abundance increases significantly under conditions such as antibiotic exposure, hypertension, prolonged hospitalization, prematurity, and inflammatory bowel disease^[9-14]^. These findings have led to the theory that the gastrointestinal tract serves as a reservoir for *K. pneumoniae*, from which the bacterium can translocate across the intestinal epithelium and disseminate to extraintestinal sites^[15-19]^.

A key barrier to microbial translocation is the intestinal mucus layer, which protects the underlying epithelium from potentially harmful microbes. Intestinal mucus is composed largely of the mucin protein Mucin 2 (MUC2), which is decorated with O-linked glycans, such as N-acetyl-neuraminic acid, fucose, galactose, N-acetyl-glucosamine (GlcNAc), and N-acetyl-galactosamine (GalNAc)^[20,21]^. Certain gut bacteria have evolved the ability to cleave and consume mucin glycans as nutrient sources, either for themselves or through cross-feeding other microbes^[20,22]^. Additionally, some bacteria use mucin binding proteins to adhere to and colonize the mucus layer^[23,24]^. *K. pneumoniae* has been identified in the mucus layer of mice and humans^[25,26]^ and Hudson *et al.* identified that *K. pneumoniae* harbors a fucose operon and that L-fucose metabolism is an important factor for *K. pneumoniae* colonization *in vivo*^[27]^. However, apart from these studies, little is known about the relationship between *K. pneumoniae* and mucus. We hypothesized that *K. pneumoniae* would be able to adhere to the mucus layer and potentially degrade mucin glycans to support its growth.

## METHODS

### Bacterial growth conditions

The following commercially available and clinical isolates of *Klebsiella pneumoniae* were used in this study: American Type Culture Collection (ATCC) 35657, ATCC 700603, CB1, F64162, 91901, and MCFM1-30. *K. pneumoniae* strains denoted with MCFM were kindly provided by Terri Ellis. All clinical isolates used in this study were fully de-identified bacterial specimens with no associated patient information or protected health identifiers. These isolates were collected as part of routine clinical care, banked, and provided to the investigators without any link to patient identity. In accordance with U.S. federal regulations [45 Code of Federal Regulations (CFR) 46] and Institutional Review Board (IRB) policy, use of de-identified microbial isolates does not constitute human subjects research and therefore did not require IRB review or approval. *K. pneumoniae* strains were streak plated onto *K. pneumoniae* specific agar plates (Millipore catalogue # 90925) containing the *Klebsiella* Selective Supplement (Millipore catalogue # 15821) and then subsequently plated on brain-heart infusion (BHI) agar plates for purity. Single colonies were used to inoculate BHI broth and grown at 37 °C in a shaking at 120 rpm in an aerobic incubator overnight before being used for downstream assays. As a control for mucus adhesion, we also grew *Limosilactobacillus reuteri* (*L. reuteri*) ATCC 6475 on De Man Rogosa Sharpe (MRS) agar and single colonies were used to inoculate MRS broth. 

### Fluorescently-tagging bacteria

*K. pneumoniae* strains were grown overnight as described above at a total volume of 5 mL. The following day, cultures were washed twice with sterile phosphate-buffered saline (PBS) and resuspended in 10 mL of sterile PBS. Carboxyfluorescein diacetate succinimidyl ester (CFDA-SE, ThermoFisher #C1157) was added to the bacteria in PBS at a final concentration of 50 µg/mL. Cultures were incubated in the dark at 37 °C aerobically for 1 h. Cultures were washed three times with sterile PBS to remove excess dye. To assess potential variability in dye incorporation across strains, baseline fluorescence was measured for each labeled culture and normalized to optical density at 600 nm (OD_600_). Fluorescence intensity per optical density (OD) unit was comparable across all *K. pneumonaie* strains tested, indicating minimal differences in CFDA-SE uptake. Labeled bacterial suspensions were subsequently adjusted to the same OD_600_ prior to use in adhesion assays to ensure equivalent input across conditions.

### Mucus preparation and binding assay

Porcine MUC2 (intestinal) mucus (MyBioSource #MBS5311023) was dialyzed in 7,000 MWCO Snakeskin**^TM^** Dialysis Tubing (ThermoFisher #68700) against water for three days to remove impurities or unbound glycans. The resulting mucus solution was lyophilized and resuspended in water before downstream use. Human intestinal mucus was collected from the mucus producing cell line HT29-MTX and MUC2 was isolated as previously described^[28]^. This mucus was also dialyzed and lyophilized. Mucus-coated 96-well plates were prepared by adding 1 mg/mL mucus in Hanks' Balanced Salt Solution (HBSS) (VWR #02-0121-0500) to non-tissue culture treated 96-well plates (VWR; Cat# 10861-562) overnight at 4 °C. As a negative control, plates were coated with 1 mg/mL of methylcellulose (Fisher Scientific #M352-500) in HBSS. After the overnight incubation, the plates were washed twice with sterile PBS. The OD_600_ of fluorescently tagged bacteria was measured to determine the concentration of bacteria. Tagged-bacteria was put to an OD_600_ = 1.0 in PBS and 100 µL was plated into respective wells of the mucus-coated 96-well plates. The plates were incubated in the dark at 37 °C for 1 h. The plates were washed twice with sterile PBS to remove unbound bacteria. The fluorescence (excitation: 485 nm; emission: 528 nm) was read to determine adhered bacteria. As confirmation of adhesion, fluorescently tagged *K. pneumoniae* and a positive control *L. reuteri* was added at an OD_600_ =2, 1, 0.5, and 0.1 to generate an adhesion curve. To examine whether *K. pneumoniae* ATCC 35657 could adhere to human intestinal mucus, fluorescently tagged *K. pneumoniae *was added at OD_600_ = 1.0 in PBS to glass coverslips (18 mm) coated with 1 mg/ml human intestinal mucus using 3-aminopropyltriethoxysilane (APTS) as previously described^[28,29]^. *K. pneumoniae* was incubated with coverslips at 37 °C for 1 h. After washing, adhesion was examined on a Zeiss Axio microscope with a 60× objective.

### Growth and biofilm assay

Bacteria were grown overnight in BHI before being sub-cultured into a M9 minimal medium (ThermoFisher #A 1374401). The M9 medium was supplemented with water, 25 mM D-glucose (FisherSci #A16828-36), L-fucose (FisherSic #A16789-03), N-acetyl-neuraminic acid (Neu5AC, FisherSci #L13950-MC), D-galactose (FisherSci # A12813-0B), GlcNAc, FisherSci # A13047-22, GalNAc, FisherSci # J66095-06, or dialyzed porcine intestinal mucus or human intestinal mucus (1, 0.5 and 0.25 mg/mL) and plated onto a non-tissue culture treated 96-well plate (VWR; Cat# 10861-562). Bacteria were added to the M9 medium at an optical density OD_600 _of 0.1. Plates were incubated at 37 °C and read on Synergy HT plate reader at OD_600_ every 10 minutes for 18 hours. For biofilm assays, we coated non-tissue culture treated 96-well plates (VWR; Cat# 10861-562) with 1 mg/mL porcine MUC2 mucus or 1 mg/mL of methylcellulose in HBSS overnight at 4 °C. After washing, we added *K. pneumoniae* at OD_600_ of 0.1 in BHI to the plates and incubated them at 37 °C for 48 h. Following the incubation, we washed the plates with PBS and stained with 0.1% crystal violet for 1 h at 37 °C. After washing, the crystal violet was solubilized in alcohol-acetone (80%/20%) solvent and the absorbance values at 570 nm were read on a Synergy H1 plate reader.

### Antibiotics resistance curve

Bacteria were grown overnight in BHI before being sub-cultured into a Luria-Bertani (LB) medium (ThermoFisher #A 1374401). The LB medium was supplemented with water or 1 mg/mL human MUC2 mucus and used to generate a serial dilution of the aminoglycoside antibiotics gentamicin, kanamycin, and streptomycin (2,048 μg/mL to 2 μg/mL). *K. pneumoniae* was added at an OD_600 _of 0.1. Plates were incubated at 37 °C and read on Synergy HT plate reader at OD_600_ every 10 min for 18 h. The half maximal inhibitory concentration (IC_50_) value was calculated for each antibiotic at the 18 h time point.

### Quantitative real time polymerase chain reaction assay

*K. pneumoniae* strains CB1 and ATCC 35657 were grown in a chemically defined minimal medium (CDMM) supplemented with either water or 1 mg/mL dialyzed porcine intestinal mucus and incubated for 16 h at 37 °C in a shaking aerobic incubator. RNA was extracted from the bacteria using the Quick-RNA MiniPrep Kit (Zymo #R1055) per the manufacturer’s instructions with the addition of bead beating. Briefly, bacterial pellets were resuspended in 100 µL STE buffer [100 mM NaCl, 10 mM Tris-HCl, pH 8.0, 1 mM ethylenediaminetetraacetic acid (EDTA)], and transferred to 1.5 mL screw-top tubes containing 0.1 mm glass beads. Bacteria were disrupted on a Bedbug bead homogenizer (Benchmark Scientific) for 20 s at 4.0 m/s, then Zymo Quick RNA lysis buffer was added to each tube, and samples were disrupted for a second time. Following these steps, RNA was isolated according to the manufacturer’s protocol. RNA quality was assessed by Nanodrop (ThermoScientific) and was then used to make cDNA with the Verso cDNA Synthesis Kit (ThermoScientific #AB1453) per the manufacturer’s instructions. Quantitative real time polymerase chain reaction (qPCR) was performed using qPCRBIO SyGreen Mix lo-ROX (PCRBiosystems #PB20.11). The data was analyzed using the ΔddCT method.

### Mouse model

All the experiments involving animals were reviewed and approved by the Institutional Animal Care and Use Committee (IACUC) at the Medical University of South Carolina (protocol #2021-01286). All experimental procedures strictly adhered to the guidelines set forth by the US National Institutes of Health (NIH) Office of Laboratory Animal Welfare (OLAW) and complied with the regulations under the Animal Welfare Act and the Public Health Service (PHS) Policy on Humane Care and Use of Laboratory animals. Male C57BL/6J mice (10 weeks of age) were purchased from Jackson Labs (#000664) and housed under standard specific pathogen-free conditions at controlled temperature and humidity (22 ± 2 °C and 40%-60% humidity) with a 12-h light/dark cycle and ad libitum access to food and water. Mice were acclimated for at least one week prior to experimentation and were randomly assigned to experimental groups. Investigators were not blinded during the experimental procedures, but were blinded during image analysis. For the experiment, the drinking water was switched with antibiotic water that contained 0.4 mg/mL kanamycin, 35 µg/mL gentamicin, 68 µg/mL colistin, 0.215 mg/mL metronidazole, and 45 µg/mL vancomycin. After 5 days, the antibiotic water was switched with standard autoclaved drinking water and mice received a single dose intraperitoneal injection of 0.25 mg of clindamycin in water. Mice were randomly assigned to two groups (*n* = 5 per group; total of 10 mice in the experiment) and were orally gavaged with sterile PBS (vehicle control) or 10^7^ colony forming units (CFU) *K. pneumoniae*. After 5 days, mice were humanely euthanized and the colon was fixed in Carnoy’s fixative. Fluorescent *in situ* hybridization (FISH) staining was performed on 5 µm thick sections of colon on glass slides. Slides were hybridized with the probe [Cy3] CACCTACACACCAGC^[30]^ (Integrated DNA Technologies, IDT) in a humidified dark chamber at 51 °C for 45 min. Slides were then washed in pre-warmed washing buffer and water and and counter-stained with the mucus glycan targeting lectin Ulex europaeus agglutinin I (UEA I-FITC; VectorLabs #FL-1061-2) for 1 h at room temperature. After washing, the slides were then stained with the nuclear stain Hoechst 33424 (FisherSci # 51-17) for 10 min at room temperature. Sections were analyzed by fluorescence microscopy on a Zeiss Axio microscope with a 20× objective. 

#### Data mining

To assess the distribution of mucus-associated glycosyl hydrolases among *K. pneumoniae* genomes, we queried the Integrated Microbial Genomes (IMG) database v5.0 (http://img.jgi.doe.gov)^[31]^, which is available through the Joint Genomes Institute (JGI) (Version 6.0) on March 26^th^, 2022. Enzyme Commission (EC) numbers for mucus-degrading enzymes were input into the gene search tool of the IMG database and *K. pneumoniae* bacterial genomes were binned for analysis. Genomes that harbored at least one gene copy of a specified EC were considered to possess that function. To determine how many *K. pneumoniae* strains possessed an EC function, we used the following equation^[32]^:

**Figure:**
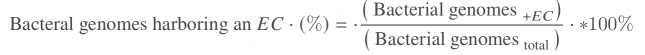


### Statistics and graphs

All graphs were generated using GraphPad Prism software (version 10.0.03) (GraphPad Inc.). Overnight growth curves and biofilm data was analyzed by Two Way ANOVA with the Tukey post-hoc test. Adhesion was analyzed by One Way Analysis of Variance (ANOVA) with the Tukey post-hoc test. IC_50_ values for antibiotic susceptibility were calculated from a four-parameter logistics curve. Each dot in the graph represents an individual data point. The data are presented as mean ± standard deviation, with *P* < 0.05 (*) considered statistically significant. 

## RESULTS

### *K. pneumoniae* adheres to intestinal mucus

**Figure 1:**
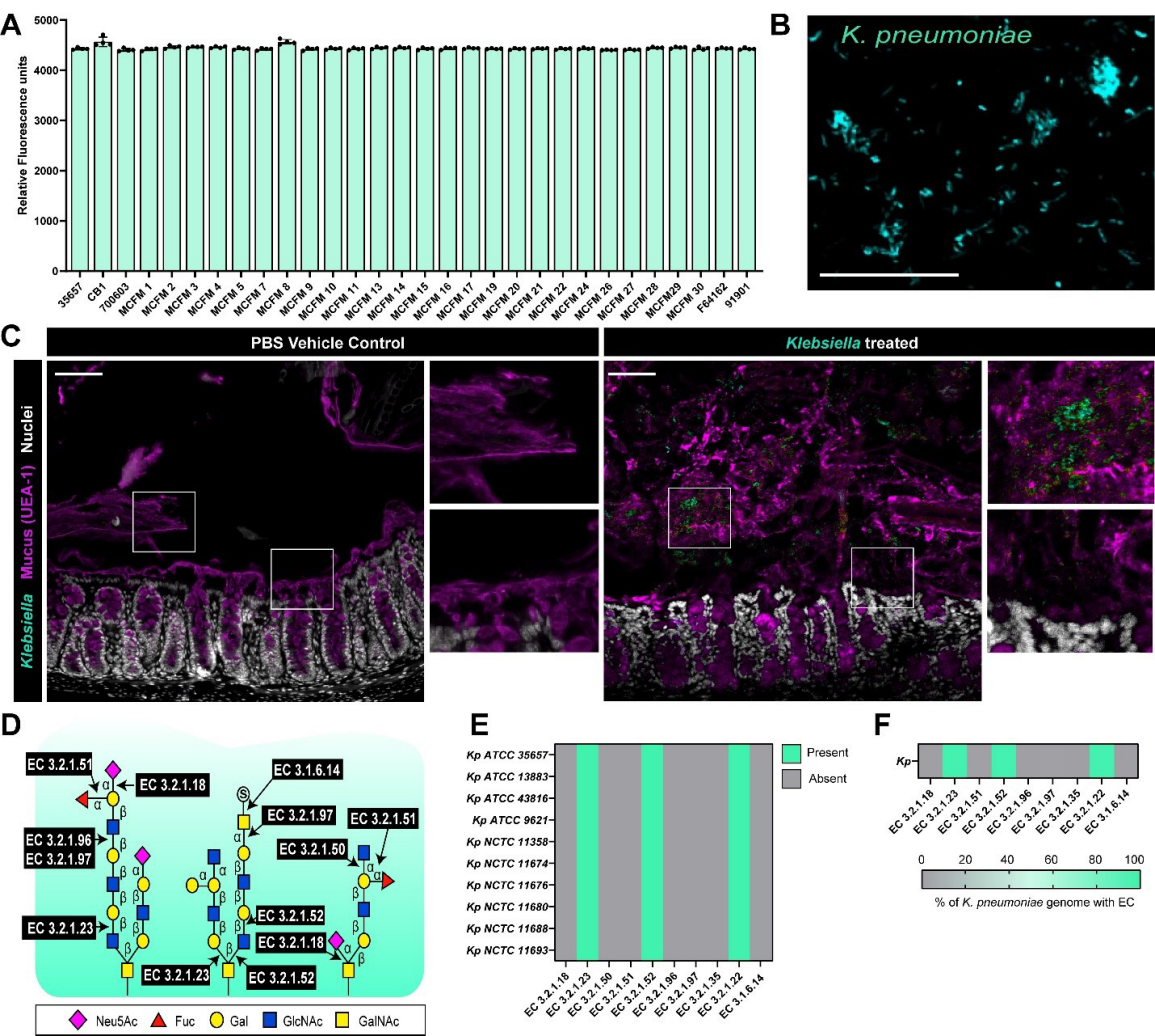
*K. pneumoniae *can adhere to intestinal mucus. (A) *K. pneumoniae* strains were fluorescently tagged and added to plates coated with 1 mg/mL dialyzed porcine intestinal mucus and adhesion was examined by a plate reader after 1 h incubation. Data presented as the fluorescence of adhered bacteria to mucus (mean ± standard deviation); (B) Representative fluorescent microscopy image of *K. pneumoniae* ATCC 35657 adhered to human MUC2-coated coverslips, scale bar = 50 μm; (C) Mice were orally administered with PBS (vehicle control) or *K. pneumoniae* ATCC 35657 and colons were harvested five days later. *K. pneumoniae* in the mucus layer was visualized with fluorescent *in-situ* hybridization (teal), mucus was visualized with UEA-1 staining (purple) and nuclei were identified with Hoechst (white). Scale bar = 50 μm; (D) Schematic of the enzymes required to cleave the bonds attaching mucus-associated sugars; (E) Genome analysis of 10 *K. pneumoniae* genomes in the IMG database for genes involved in mucus glycan degradation; (F) Genome analysis of the percentage of total *K. pneumoniae* genomes in the IMG database for genes involved in mucus glycan degradation. Data represents the percentage of genomes with a given EC. Data represents the presence or absence of the target gene. Graphs generated with Graphpad Prism. *K. pneumoniae*: *Klebsiella pneumoniae*; PBS: phosphate-buffered saline; EC: Enzyme Commission; ATCC: American Type Culture Collection; MUC2: Mucin 2; IMG: Integrated Microbial Genomes; UEA-1: Ulex europaeus agglutinin I.

To assess the ability of *K. pneumoniae* to adhere to intestinal mucus, we fluorescently tagged multiple commercially available (ATCC 35657, ATCC 700603, CB1,) and clinical isolates (MCFM1-30, F64162, 91901) of *K. pneumoniae* and examined adhesion to porcine MUC2 mucus. We found that all the *K. pneumoniae* strains adhered to mucus [Figure 1A], suggesting that mucus adhesion is a conserved feature among *K. pneumoniae *strains. As additional confirmation, we serially diluted *K. pneumoniae* and a positive control *L。 reuteri*, which is known to adhere to mucus^[33-35]^, on mucus coated plates. We found that both *L. reuteri* and *K. pneumoniae* exhibited a dose-dependent adhesion to porcine MUC2 mucus [Supplementary Figure 1A and B]. As a negative control we also examined the adhesion of *K. pneumoniae* to methylcellulose; a polymer that is commonly used to mimic the viscosity of mucus, but lacks the glycans for specific adhesion^[28]^. Although we did note some non-specific adhesion to methylcellulose, the adhesion was far lower than observed for mucus [Supplementary Figure 1C]; suggesting that *K. pneumoniae* adheres to mucus. To confirm that *K. pneumoniae* was capable of binding to human intestinal mucus, we added fluorescently tagged *K. pneumoniae *ATCC 35657 to human MUC2 on glass-coated coverslips [Figure 1B]. Microscopy revealed robust bacterial adhesion, with numerous *K. pneumoniae* cells bound to the MUC2 mucus. To assess if *K. pneumoniae* was adept at adhering to mucus *in vivo*, we oral gavaged adult C57B6/J mice with either PBS (vehicle control) or *K. pneumoniae *ATCC 35657 and examined mucus adhesion by FISH. As expected, we saw no *K. pneumoniae* in the PBS treated animals, but we found *K. pneumoniae* adhered to the mucus layer above the epithelium in the *K. pneumoniae* treated animals [Figure 1C]. These data indicate that *K. pneumoniae* adheres to intestinal mucus *in vitro* and *in vivo*. 

### *K. pneumoniae *possesses glycosyl hydrolases necessary for limited mucus degradation

The mucin protein is heavily glycosylated and contains chains of sugars such as galactose, GlcNAc, GalNAc that are terminated by fucose and N-acetyl-neuraminic acid^[20]^. To identify if *K. pneumoniae* had the genes necessary for cleaving the glycan linkages in mucus [Figure 1D], we examined 1,052 annotated *K. pneumoniae* genomes on the Joint Genome Institute’s IMG database and queried the number of genomes that possessed the glycosyl hydrolases required for mucus glycan degradation [Figure 1E]. We first examined the genomes of commercially available *K. pneumoniae* strains and found that all the strains possessed at least one gene copy of β-galactosidases (EC 3.2.1.23), and α-galactosidases (EC 3.2.1.22); suggesting that these bacteria could remove galactose residues [Figure 1E]. We also found that these *K. pneumoniae* strains possessed β-N-acetyl-hexosaminidases (EC 3.2.1.52), which remove GlcNAc and GalNAc residues. We found a similar pattern in the > 1,000 annotated *K. pneumoniae* genomes, with ~ 98% of *K. pneumoniae* genomes having β-galactosidases (EC 3.2.1.23), α-galactosidases (EC 3.2.1.22), and β-N-acetyl-hexosaminidases (EC 3.2.1.52) [Figure 1F]. Interestingly, none of the *K. pneumoniae* strains possessed fucosidases (EC 3.2.1.51), neuraminidases (EC 3.2.1.18) or sulfatases (EC 3.1.6.14). These data suggest that *K. pneumoniae* encode enzymes for cleaving internal glycan sugars such as galactose, GlcNAc and GalNAc residues, but lacks the enzymes necessary to remove terminal fucose, N-acetyl-neuraminic acid, or sulfate groups.

### *K. pneumoniae *possesses glycosyl transferases necessary for the uptake of mucus associated sugars

**Figure 2:**
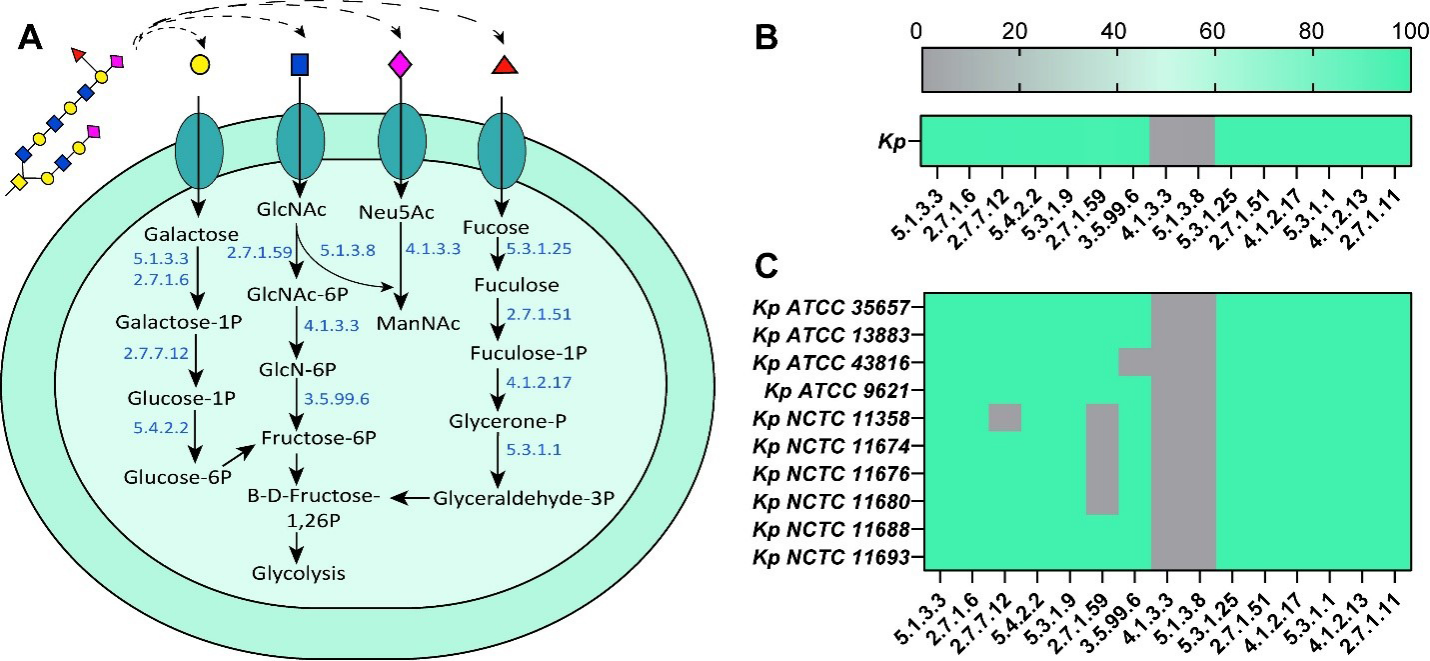
*K. pneumoniae *possesses multiple enzymes necessary for mucus-associated sugar metabolism. (A) Schematic of the necessary genes for the down-stream processing of mucus-associated sugars; (B) Genome analysis of the percent of *K. pneumoniae* genomes in the IMG database for the presence sugar processing associated genes; (C) Genome analysis of 10 *K. pneumoniae* genomes in the IMG database for the presence of sugar-processing associated genes. Data represents the presence or absence of the target gene. Graphs generated with Graphpad Prism. GlcNAc: N-acetyl-glucosamine; Neu5Ac: N-acetyl-neuraminic acid; ATCC: American Type Culture Collection; NCTC: National Collection of Type Cultures; *K. pneumoniae*: *Klebsiella pneumoniae*; IMG: Integrated Microbial Genomes.

We next examined if *K. pneumoniae* possessed the downstream pathways required for processing mucin-derived sugars. Using the IMG database, we queried *K. pneumoniae* genomes for key sugar utilization pathways [Figure 2A]. Overall, the *K. pneumoniae* genomes encoded numerous enzymes involved in processing galactose, GlcNAc, fructose, and fucose metabolism, including D-galactose-1-phosphotransferase (EC 2.7.1.6), α-D-galactose-1-phosphate uridylyltransferase (EC 2.7.7.12), N-acetyl-D-glucosamine 6-phosphotransferase (EC 2.7.1.59), β-D-fructose-6-phosphate 1-phosphotransferase (EC 2.7.1.11), fructose-bisphosphate aldolase (EC 4.1.2.13), L-fuculose-phosphate aldolase (EC 4.1.2.17), glucose-6-phosphate isomerase (EC 5.3.1.9), and α-D-glucose-1,6-phosphomutase (EC 5.4.2.2) [Figure 2B]. Analysis of the commercially available strains mirrored this pattern, with most strains harboring the same metabolic pathways identified across the > 1,000 genomes [Figure 2C]. At the individual strain level, however, we noted some variation; for example, *K. pneumoniae* National Collection of Type Cultures (NCTC) 11358 lacked several enzymes required for converting galactose-1-phosphate to glucose-1-phosphate and GlcNAc-6-phosphate to GlcN-6-phosphate [Figure 2C]. None of *K. pneumoniae* genomes had EC 5.1.3.8 to convert GlcNAc to N-Acetylmannosamine (ManNAc) or EC 4.1.3.3 to convert N-acetyl-neuraminic acid (Neu5Ac) (sialic acid) to ManNAc. Together, these findings indicate that *K. pneumoniae* broadly encodes the metabolic capacity to utilize liberated mucin-derived sugars, although specific pathway gaps may contribute to strain-level differences in sugar utilization.

### *K. pneumoniae *can use individual mucin glycans, but not intact mucus, for growth

**Figure 3:**
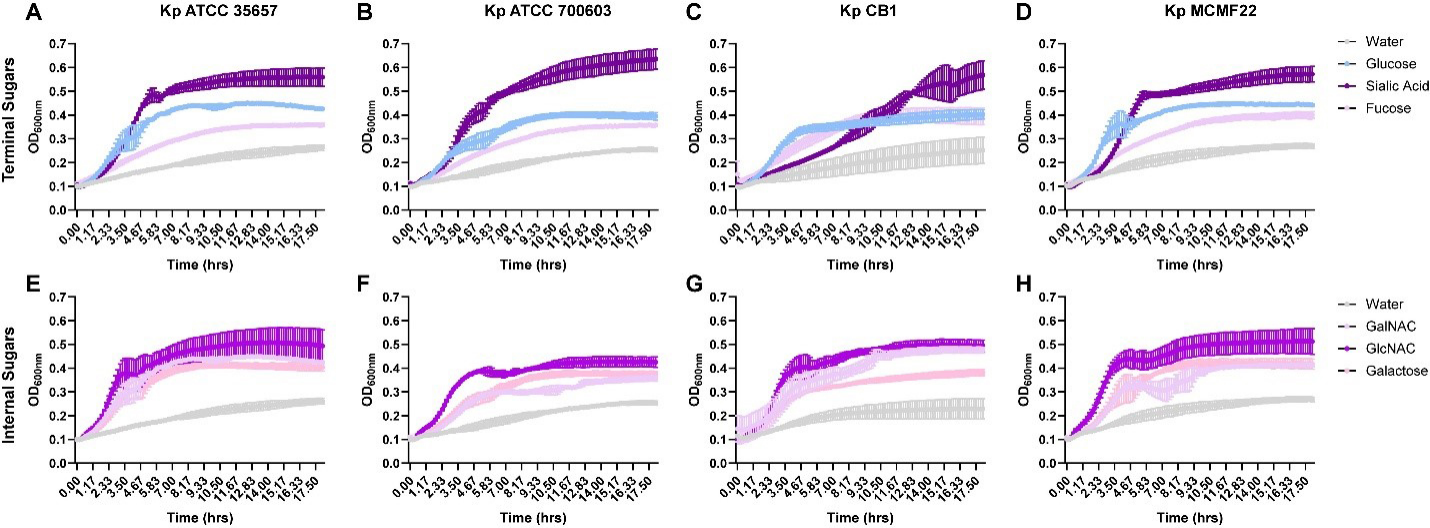
*K. pneumoniae *can use mucus-associated sugars as a fuel source. *K. pneumoniae* ATCC 35657 (A and E), ATCC 700603 (B amd F), CB1 (C and G), and MCFM22 (D and H) were grown in a M9 minimal medium supplemented with (A-D) 25 mM D-glucose, N-acetyl-neuraminic acid, L-fucose or (E-H) 25 mM D-galactose, GlcNAc, and GalNAc for 18 h. The OD_600_ was measured by plate reader to monitor growth over time. Data are represented as mean ± standard deviation. Graphs generated with Graphpad Prism. *K. pneumoniae*: *Klebsiella pneumoniae*; ATCC: American Type Culture Collection; GlcNAc: N-acetyl-glucosamine; GalNAc: N-acetyl-galactosamine; OD_600_: optical density at 600 nm.

We next tested whether *K. pneumoniae* could utilize mucus-associated sugars as a fuel source. To do this, we grew *K. pneumoniae* ATCC 35657, ATCC 700603, CB1, and MCFM22 in M9 minimal medium supplemented with 25 mM of individual mucin-derived monosaccharides and monitored growth over time. As a positive control for carbon utilization, we included 25 mM glucose. As expected, we found that all *K. pneumoniae* strains had enhanced growth with our positive control glucose [Figure 3A-D and Table 1]. We found that 25 mM of the terminal glycan sugars N-acetyl-neuraminic acid and L-fucose were able to significantly increase *K. pneumoniae* growth throughout the exponential and lag phase [Figure 3A-D]. We likewise found that 25 mM of the internal glycan sugars galactose, GalNAc and GlcNAc promoted an increase in growth during exponential and lag phase of all our *K. pneumoniae* strains when compared to the water control [Figure 3E-H and Table 1]. 

**Table 1:** Statistics of *K. pneumoniae *strains grown in M9 minimal medium with 25 mM mucin glycan sugars

**Comparison**	**Kp ATCC 35657**	**Kp ATCC 700603**	**Kp CB1**	**Kp MCFM22**
Water *vs.* Glucose	< 0.0001	< 0.0001	< 0.0001	< 0.0001
Water *vs.* Galactose	< 0.0001	< 0.0001	< 0.0001	< 0.0001
Water *vs.* GalNAc	< 0.0001	< 0.0001	< 0.0001	< 0.0001
Water *vs.* GlcNAc	< 0.0001	< 0.0001	< 0.0001	< 0.0001
Water *vs.* Fucose	0.0009	0.0001	< 0.0001	< 0.0001
Water *vs.* Neu5Ac	< 0.0001	< 0.0001	< 0.0001	< 0.0001

Data represents the *P* values at the final 18 h time point as assessed by a Two Way ANOVA. *K. pneumoniae*: *Klebsiella pneumoniae*; ATCC: American Type Culture Collection; GalNAc: N-acetyl-galactosamine; Neu5Ac: N-acetyl-neuraminic acid; ANOVA: Analysis of Variance.

**Figure 4:**
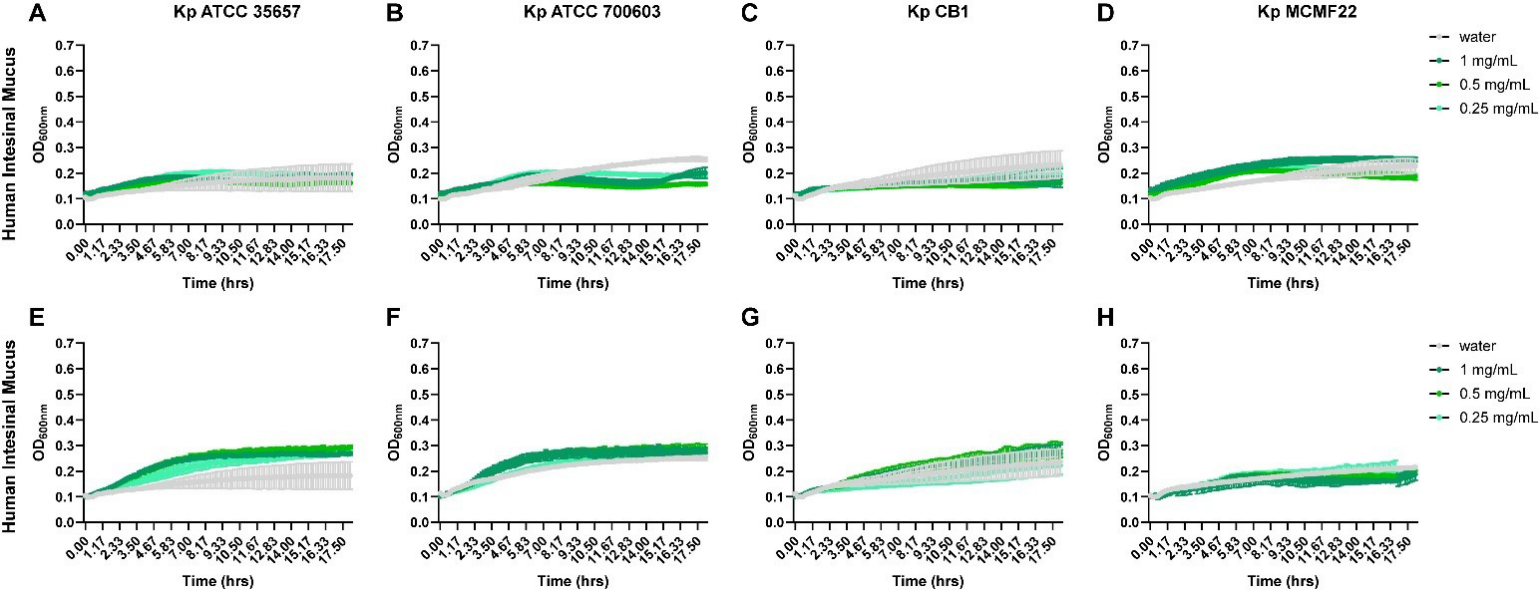
*K. pneumoniae* is unable to use intact mucus as a fuel source. *K. pneumoniae* ATCC 35657 (A and E), ATCC 700603 (B and F), CB1 (C and G), and MCFM22 (D and H) were grown in a M9 minimal medium supplemented with (A-D) porcine MUC2 (1, 0.5, and 0.25 mg/mL) or (E and H) human MUC2 (1, 0.5, and 0.25 mg/mL) for 18 h. The OD_600_ was measured by plate reader to monitor growth over time. Data are represented as mean ± standard deviation. Graphs generated with Graphpad Prism. ATCC: American Type Culture Collection; OD_600_: optical density at 600 nm; *K. pneumoniae*: *Klebsiella pneumoniae*; MUC2: Mucin 2.

We next tested if *K. pneumoniae* could utilize intact mucus as a fuel source by growing our four strains of *K. pneumoniae* in M9 supplemented with different concentrations of porcine MUC2 mucus [Figure 4A-D and Table 2] or human MUC2 mucus [Figure 4E-H and Table 2]. None of the *K. pneumoniae* strains could use porcine or human MUC2 to support growth [Figure 4]. These data suggests that while *K. pneumoniae* can metabolize liberated mucin sugars, it lacks the enzymes required to remove terminal glycan residues and thus cannot substantially degrade mucus and consume the released glycans.

**Table 2:** Statistics of *K. pneumoniae* strains grown in M9 minimal medium with porcine or human MUC2

**Mucus type**	**Comparison**	**Kp ATCC 35657**	**Kp ATCC 700603**	**Kp CB1**	**Kp MCFM22**
Porcine MUC2	Water *vs.* 1 mg/mL	> 0.9999	0.2129	0.1137	0.9998
Porcine MUC2	Water *vs.* 0.5 mg/mL	> 0.9999	0.1664	0.1001	0.9992
Porcine MUC2	Water *vs.* 0.25 mg/mL	> 0.9999	0.2006	0.125	0.9994
Human MUC2	Water *vs.* 1 mg/mL	0.9996	0.9991	> 0.9999	0.9966
Human MUC2	Water *vs.* 0.5 mg/mL	> 0.9999	> 0.9999	> 0.9999	0.9971
Human MUC2	Water *vs.* 0.25 mg/mL	0.9998	0.9996	0.9995	> 0.9999

Data represents the *P* values the final 18 h time point as assessed by a Two Way ANOVA. *K. pneumoniae*: *Klebsiella pneumoniae*; MUC2: Mucin 2; ATCC: American Type Culture Collection; ANOVA: Analysis of Variance.

### The presence of mucus does not impact biofilm formation or virulence genes, but it does alter *K. pneumoniae* antibiotic resistance

To determine whether mucus influences *K. pneumoniae* community behavior, we next examined biofilm formation in the presence and absence of porcine MUC2. Although we observed strain-dependent differences in baseline biofilm production, with notably higher biofilm formation by CB1 and MCFM22 compared to ATCC 35657 and ATCC 700603, the addition of mucus did not alter biofilm levels for any strain [Supplementary Figure 1D]. These findings indicate that while biofilm capacity varies across *K. pneumoniae* isolates, mucus does not modulate biofilm production under these conditions. We next sought to determine whether mucus alters the expression of well-characterized *K. pneumoniae* virulence factors. *K. pneumoniae* CB1 and ATCC 35657 were grown in the presence or absence of porcine MUC2, and gene expression was assessed by qPCR [Supplementary Figure 2A-L]. The presence of mucus did not significantly alter the expression of genes associated with iron acquisition (*iroD*), urease production (*ureA*), type 1 fimbriae (*fimH*), fatty acid biosynthesis (*clbA*, *clbB*), or allantoin metabolism (*allS*). These findings suggest that mucus does not impact canonical virulence pathways. Finally, we examined the impact of mucus on antibiotic resistance. *K. pneumoniae* is well recognized for its intrinsic resistance to many antibiotics and its capacity to rapidly acquire additional resistance determinants^[2,8,36,37]^. We examined growth with the antibiotics gentamicin, kanamycin and streptomycin. Across all four *K. pneumoniae* strains tested, and the presence of human MUC2 consistently lowered the IC_50_ values for gentamicin, kanamycin and streptomycin, indicating increased antibiotic sensitivity in the presence of mucus [Table 3]. For gentamicin, IC_50_ values decreased by approximately 45%-65% across strains, with ATCC 35657 dropping from 5.6 to 2.6 µg/mL and MCFM22 from 6.6 to 2.4 µg/mL. A similar trend was observed for kanamycin and streptomycin, where IC_50_ values declined by ~ 25%-55% and ~ 20%-60% respectively. These results demonstrate that mucus does not protect *K. pneumoniae* from antibiotic activity; instead, mucus enhances susceptibility to aminoglycosides across diverse strains.

**Table 3:** Calculated IC_50_ values for *K. pneumoniae* strains grown in LB with water (control) or human MUC2 mucus with the antibiotics gentamicin, kanamycin and streptomycin

	**Gentamicin**		**Kanamycin**		**Streptomycin**	
**Strain**	**Control**	**MUC2**	**Control**	**MUC2**	**Control**	**MUC2**
ATCC 35657	5.64	2.56	10.80	7.97	8.21	2.43
ATCC 700603	7.00	2.97	18.09	10.50	3.09	1.84
CB1	7.57	3.78	11.16	5.13	7.98	5.05
MCMF22	6.63	2.41	17.95	13.66	5.01	4.45

IC_50_: Half maximal inhibitory concentration; LB: Luria-Bertani; MUC2: Mucin 2; ATCC: American Type Culture Collection.

## DISCUSSION

In this study, we evaluated how *K. pneumoniae* strains interact with intestinal mucus and how this interaction influences bacterial behavior. We found that multiple *K. pneumoniae* strains adhered robustly to porcine and human MUC2, and that *K. pneumoniae* ATCC 35657 also localized to the mucus layer in the mouse colon *in vivo*, demonstrating that mucus adhesion is a conserved trait. Genomic analyses of more than 1,000 *K. pneumoniae* genomes revealed that the species broadly encodes glycosyl hydrolases capable of processing internal mucin-derived sugars. However, *K. pneumoniae* lacks enzymes required to cleave terminal fucose, N-acetyl-neuraminic acid, and sulfate groups and was unable to grow on intact MUC2 as a sole carbon source, indicating a limited capacity for direct mucin degradation. Consistent with the *K. pneumoniae* genomic profile, all strains readily used free mucin-derived monosaccharides, including galactose, GlcNAc, GalNAc, fucose, and N-acetyl-neuraminic acid; highlighting a reliance on liberated sugars rather than intact mucin glycans. Although strains varied in their baseline biofilm formation, the presence of mucus did not alter biofilm production for any strain. Unexpectedly, however, exposure to MUC2 increased *K. pneumoniae* susceptibility to the aminoglycosides gentamicin, kanamycin and streptomycin across all strains tested. Together, these findings establish that while *K. pneumoniae* efficiently adheres to intestinal mucus and exploits available mucin-derived sugars, intact mucus enhances antibiotic sensitivity, revealing mucus as an important modulatory factor in *K. pneumoniae* colonization dynamics and treatment response.

While *K. pneumoniae* is not classically considered a predominant member of the gut microbiota^[38-41]^, it has been shown to be elevated during different intestinal disease states^[16,42-48]^, such as inflammatory bowel disease (IBD)^[49-53]^ and *Clostridioides difficile* infection^[54,55]^. A common feature of these conditions is a disrupted microbial community, and the loss of key commensal organisms likely creates ecological niches that allow opportunistic bacteria such as *K. pneumoniae* to expand^[44,56]^. In the absence of microbial competitors, we propose that *K. pneumoniae* can bind to the intestinal mucus layer and utilize mucus-derived nutrients to support its growth. Despite possessing a limited repertoire of mucin-degrading enzymes, *K. pneumoniae* may benefit from cross-feeding by classic mucin degraders, such as *Bacteroides thetaiotaomicron*, *Ruminococcus torques*, or *Akkermansia muciniphila*^[20]^. Such cooperative interactions are well documented in the gut^[57,58]^ and may similarly facilitate *K. pneumoniae* persistence within the mucus layer. Although this model requires further investigation, these potential interactions have important clinical implications for patients experiencing microbiota disruption. 

N-acetyl-neuraminic acid import and utilization is important for a variety of enteric pathogens^[58-61]^. An interesting observation from our study was that *K. pneumoniae* did not encode the enzymes required to convert Neu5Ac to ManNAc (EC 4.1.3.3), yet N-acetyl-neuraminic acid still supported bacterial growth. This could be because *K. pneumoniae* is able to import N-acetyl-neuraminic acid and channel it into alternative metabolic pathways independent of the canonical ManNAc route. Several bacteria, including *E. coli, Salmonella enterica*, and* Vibrio *species can import N-acetyl-neuraminic acids through NanT-like transporters^[62-66]^. Inside bacteria, N-acetyl-neuraminic acid can bind to NanR and influence transcription^[67,68]^. Although Nan clusters have not been previously reported in *K. pneumoniae*, it is possible that N-acetyl-neuraminic acid may act as an environmental signal to modulate gene expression in *K. pneumoniae*.

Biofilm formation is a key virulence factor for *K. pneumoniae*, contributing to persistence, antibiotic resistance, and host colonization^[69-76]^. Many environmental cues have been shown to enhance *K. pneumoniae* including low iron availability, limited nutrients, and low-oxygen conditions^[77-80]^. Mucus is known to impact biofilm production on diverse microbes. For example, mucus enhances biofilm production by the gut pathobiont *Clostridioides difficile*^[28,81]^ and decreases biofilm production by the respiratory pathobiont *Pseudomonas aeruginosa*^[82,83]^. In this study, we found that mucus did not significantly alter biofilm formation by *K. pneumoniae*. This suggests that while mucus provides an adhesive substrate, it does not inherently promote biofilm development under the conditions tested. Instead, *K. pneumoniae* may rely on other environmental cues, host factors, or microbial signals to modulate biofilm architecture within the intestinal environment. 

One of the most unexpected observations in this study was that mucus increased *K. pneumoniae* sensitivity to gentamicin, kanamycin, and streptomycin across all strains tested. Aminoglycoside resistance is a hallmark of *K. pneumoniae*, driven by intrinsic mechanisms and rapid acquisition of resistance determinants^[84-86]^. Several compounds that are found in the gut could impact *K. pneumoniae’*s resistance to antibiotics. Antimicrobial peptides are generated by Paneth and goblet cells in the small intestine and goblet cell in the large intestine^[87,88]^ and antimicrobial peptides have been shown to synergize with antibiotics like chloramphenicol, meropenem, rifampicin, or ceftazidime to effectively kill multidrug resistant *K. pneumoniae* isolates^[89]^. Additionally, short chain fatty acids that are produced by commensal microbes can synergize with antibiotics like cefoperazone and sulbactam to inhibit Enterobacteriaceae member *Escherichia coli*^[90]^. We found that mucus is another gut factor that can alter antibiotic resistance in *K. pneumoniae*. We observed a consistent reduction in IC_50_ values for gentamicin, kanamycin, and streptomycin in the presence of human MUC2, suggesting that mucus alters *K. pneumoniae* physiology in a way that enhances aminoglycoside efficacy. One possibility is that mucus serves as a colonization-associated signal, prompting *K. pneumoniae* to shift its gene expression toward adhesion or nutrient acquisition pathways, inadvertently increasing susceptibility to antibiotics. This observation may have therapeutic implications in clinical contexts where mucus could be used to potentially enhance antibiotic susceptibility in *K. pneumoniae*.

Another potential explanation for the increased aminoglycoside sensitivity observed in the presence of mucus is that mucus alters antibiotic uptake in *K. pneumoniae*. Aminoglycosides require active transport across the bacterial inner membrane, a process that is highly dependent on the proton motive force and cellular respiration proton motive force^[84-86,91,92]^. Environmental factors that enhance respiration or membrane potential can increase aminoglycoside uptake and bactericidal activity^[91,93,94]^. Mucus could shift bacterial metabolism toward a more energetically active state, thereby increasing proton motive force-dependent antibiotic uptake. Additionally, mucin glycans could be impacting antibiotic efflux. *K. pneumoniae* harbors several multidrug efflux transporters that allow these organisms to evade antibiotics^[95-98]^. Compounds that target these efflux transporters have been shown to improve antibiotic driven killing of *K. pneumoniae*^[98-101]^. It is unclear how mucus may be facilitating this, but it is possible that mucus suppresses efflux transporters and thus promotes antibiotic susceptibility. Future studies examining antibiotic resistance pathways are warranted to better understand this phenomenon.

Several limitations should be considered when interpreting these findings. First, while our *in vitro* assays provided controlled insights into mucus adhesion, sugar utilization, and antibiotic sensitivity, they cannot fully recapitulate the spatial complexity, dynamic mucus turnover, and host factors present *in vivo*. Second, we examined a subset of *K. pneumoniae* strains; although these represented both commercial and clinical isolates, additional genomic diversity, especially hypervirulent or carbapenem-resistant strains, may reveal distinct mucus-interaction phenotypes. Third, mucus composition varies across species, intestinal regions, and disease states, and using purified MUC2 may not fully reflect the heterogeneous mucus environment encountered by bacteria* in vivo*. Finally, our antibiotic assays focused on aminoglycosides, and it remains unknown whether mucus similarly affects susceptibility to other antibiotic classes. Addressing these limitations in the future will deepen our understanding of how *K. pneumoniae* interacts with the mucus barrier and how these interactions shape gut colonization and infection dynamics.

In conclusion, our findings provide new insight into the ability of *K. pneumoniae* to bind to mucus and use mucus associated sugars. These interactions shape key aspects of *K. pneumoniae* physiology, including susceptibility to aminoglycoside antibiotics. Together, our findings highlight that intestinal mucus is an active modulator of *K. pneumoniae* behavior, with the capacity to influence both microbial ecology and therapeutic responsiveness. Understanding how mucus-derived signals and microbial community dynamics converge to regulate *K. pneumoniae* fitness will be critical for developing strategies to prevent gut colonization, limit pathogen expansion during dysbiosis, and enhance antibiotic efficacy in clinical settings in the future.

## DECLARATIONS

### Availability of data and materials

The raw data supporting the conclusions of this manuscript will be made available by the authors upon request.

### Copyright

© The Author(s) 2026.
