# Supplementary material for: Intestinal mucus acts as a nutrient source and signal for Klebsiella pneumoniae
Source: Microbiome Res Rep. 2026 Apr 29;5(2):9. doi: 10.20517/mrr.2025.112 (PMC13246492; doi:10.20517/mrr.2025.112)
Supplement: Supplementary file 1 [file mrr-5-2-9-SupplementaryMaterials.pdf]

Supplementary Materials

Intestinal mucus acts as a nutrient source and signal for *Klebsiella pneumoniae*

Taylor D. Ticer[1], Pramita Suresh[2], Subhomitra Ghoshal[2], Anna M. Tingler[2], Rachel Stubler[2], Adelaide E. Horvath[2,3,4], Janiece S. Glover[2], Terri N. Ellis[5], Melinda A. Engevik[1,2]

[1]Department of Microbiology & Immunology, Medical University of South Carolina, Charleston, SC 29425, USA.
[2]Department of Regenerative Medicine & Cell Biology, Medical University of South Carolina, Charleston, SC 29425, USA.
[3]Department of Biology & Biochemistry, University of Houston, Houston, TX 77004, USA.
[4]Department of Mathematics, University of Houston, Houston, TX 77004, USA.
[5]Department of Biology, University of North Florida, Jacksonville, FL 32224, USA.

Correspondence to: Assoc. Prof. Melinda A. Engevik, Department of Regenerative Medicine & Cell Biology, Medical University of South Carolina, Charleston, SC 29425, USA. E-mail: engevik@musc.edu

ORCID: Taylor D. Ticer (0000-0002-8157-0990), Anna M. Tingler (0009-0006-8851-7080), Rachel Stubler (0000-0003-1487-1715), Adelaide E. Horvath (0009-0003-7209-8414), Janiece S. Glover (0000-0002-5781-5572), Terri N. Ellis (0000-0003-2519-7391), Melinda A. Engevik (0000-0002-9742-9932)

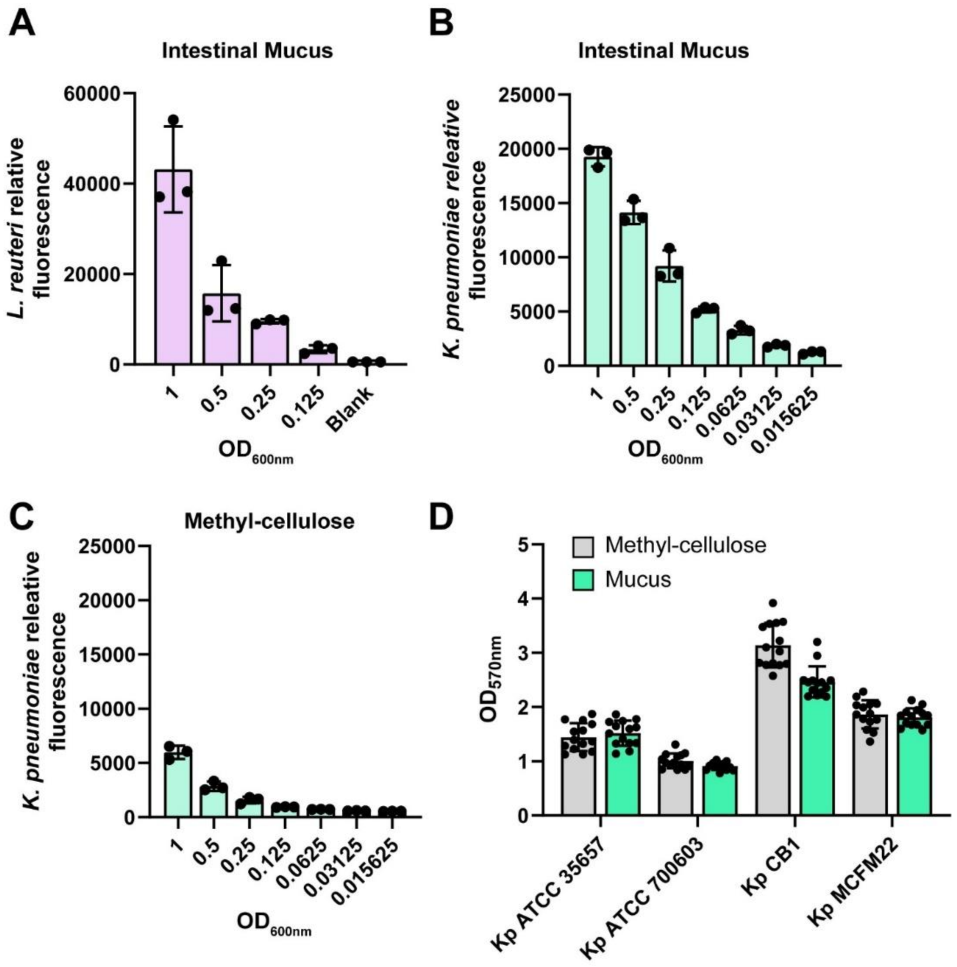

Supplemental Figure 1. *K. pneumoniae and L. reuteri adhere to mucus in a dose-dependent manner.* (A) *L. reuteri* ATCC 6475 was fluorescently tagged, adjusted to OD_{600} = 1 to 0.125 and added to plates that were coated with 1 mg/mL porcine MUC2 mucus; (B and C) *K. pneumoniae* ATCC 35657 was fluorescently tagged, adjusted to OD_{600} = 1 to 0.015 and added to plates that were coated with (B) 1 mg/mL porcine MUC2 mucus or (C) 1 mg/mL methycellulose. After 1 hour of incubation, fluorescence was measured via plate reader (excitation: 485 nm; emission: 528 nm); (D) Biofilm production of *K. pneumoniae* ATCC 35657, ATCC 700603, CB1 and MCFM22 as assessed by crystal violet staining after 48 h of incubation. Data are represented as mean ± standard deviation. Graphs generated with Graphpad Prism.

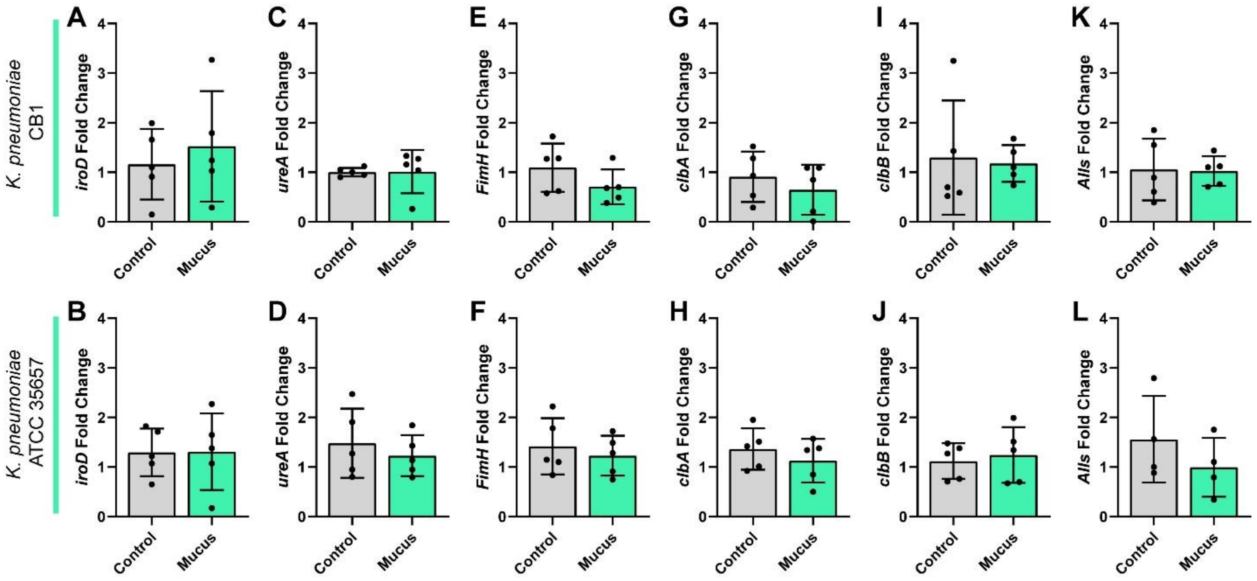

Supplemental Figure 2. Quantitative real-time PCR analysis of *K. pneumoniae* CB1 and ATCC 35657 grown in the presence of water (control) or 1 mg/mL dialyzed porcine MUC2 (mucus) for 16 hours. Gene expression is presented as fold change calculated by the ΔΔCt method. Expression of virulence-associated genes is shown for (A and B) *iroD*, (C and D) *ureA*, (E and F) *fimH*, (G and H) *clbA*, (I and J) *clbB*, and (K and L) *allS*. Data are presented as mean ± standard deviation.
